# Adapting an intervention of brief problem-solving therapy to improve the health of women with antenatal depressive symptoms in primary healthcare in rural Ethiopia

**DOI:** 10.1186/s40814-022-01166-1

**Published:** 2022-09-09

**Authors:** Tesera Bitew, Roxanne Keynejad, Bronwyn Myers, Simone Honikman, Katherine Sorsdahl, Charlotte Hanlon

**Affiliations:** 1 Department of Psychology, Injibara University, Institute of Educational and Behavioural Sciences, Injibara, Ethiopia; 2 Department of Psychiatry, Addis Ababa University, College of Health Sciences, School of Medicine, Addis Ababa, Ethiopia; 3 Section of Women’s Mental Health, King’s College London, Institute of Psychiatry, Psychology & Neuroscience, London, UK; 4 Division of Addiction Psychiatry, Department of Psychiatry & Mental Health, University of Cape Town, Cape Town, South Africa; 5 Alan J. Flisher Centre for Public Mental Health, Department of Psychiatry and Mental Health, University of Cape Town, Cape Town, South Africa; 6 Curtin enAble Institute, Faculty of Health Sciences, Curtin University, Bentley, Western Australia Australia; 7 Alcohol, Tobacco and Other Drug Research Unit, South African Medical Research Council, Cape Town, South Africa; 8 Perinatal Mental Health Project, Department of Psychiatry and Mental Health, University of Cape Town, Cape Town, South Africa; 9 Health Service and Population Research Department, Centre for Global Mental Health, King’s College London, Institute of Psychiatry, Psychology and Neuroscience, London, UK; 10 Centre for Innovative Drug Development and Therapeutic Trials for Africa (CDT-Africa), College of Health Sciences, Addis Ababa University, Addis Ababa, Ethiopia

**Keywords:** Adaptation, Problem-solving therapy, Feasibility study, Antenatal depression, Africa

## Abstract

**Background:**

Evidence-based brief psychological interventions are safe and effective for the treatment of antenatal depressive symptoms. However, the adaptation of such interventions for low- and middle-income countries has not been prioritised. This study aimed to select and adapt a brief psychological intervention for women with antenatal depressive symptoms attending primary healthcare (PHC) in rural Ethiopia.

**Methods:**

We employed the Medical Research Council (MRC) framework for the development and evaluation of complex interventions. Alongside this, we used the ADAPT-ITT model of process adaptation and the ecological validity model (EVM) to guide content adaptation. We conducted formative work, comprising a qualitative study, a series of three participatory theories of change workshops and an expert adaptation workshop to assess the needs of the target population and to select an intervention for adaptation. The adaptation process followed a series of steps: (1) training Ethiopian mental health experts in the original South African problem-solving therapy (PST version 0.0) and an initial adaptation workshop leading to PST Version 1.0. (2) Version 1.0 was presented to perinatal women and healthcare professionals in the form of a ‘theatre test’, leading to further adaptations (version 2.0). (3) Local and international stakeholders reviewed version 2.0, leading to version 3.0, which was used to train 12 PHC staff using clinical cases. (4) Finally, feedback about PST version 3.0 and its delivery was obtained from PHC staff.

**Results:**

In the first step, we modified case examples and terminology from the South African model, introduced an in-session pictorial flipchart for this low literacy setting, and added strategies to facilitate women’s engagement before translating into Amharic. In the second step, adaptations included renaming of the types of problems and inclusion of more exercises to demonstrate proposed coping strategies. In the third step, the components of motivational interviewing were dropped due to cultural incongruence. In the final step, refresher training was delivered as well as additional training on supporting control of women’s emotions to address PHC staff training needs, leading to the final version (version 4.0).

**Conclusion:**

Using a series of steps, we have adapted the content and delivery of brief PST to fit the cultural context of this setting. The next step will be to assess the feasibility and acceptability of the intervention and its delivery in antenatal care settings.

**Supplementary Information:**

The online version contains supplementary material available at 10.1186/s40814-022-01166-1.

## Key messages regarding feasibility

- In Ethiopia, mental healthcare remains highly centralised. Mental health specialists are located in urban hospitals, whereas over 80% of the population lives in rural areas. There is a large treatment gap (96% of women had no access to treatment) for perinatal depression since the focus of specialist mental healthcare is on severe mental illnesses. Expanding access to mental healthcare for women with common mental disorders, such as depression, is nationally underway through the integration of mental health into primary healthcare (PHC) and maternal care services. However, efforts to improve perinatal mental health are hampered by a lack of contextually relevant and scalable therapeutic options.
- Using a rigorous, iterative and participatory process, we selected and adapted brief problem-solving therapy to meet the needs of pregnant women with depressive symptoms in a rural, low-literacy Ethiopian context.
- The next step will be to assess the feasibility and acceptability of intervention delivery within routine antenatal care settings through a pilot randomised trial.

## Background

Untreated depression in the perinatal period is a major global public health concern. It is linked to functional impairment, reduced self-care [1, 2], suicide [3], food insecurity [4], increased patient healthcare costs [1, 5, 6], poor perinatal outcomes (e.g. low birth weight [7, 8], stillbirth [9]), delayed initiation of breastfeeding [10], poor attachment [11], poor infant health, malnutrition and delayed infant and child development [10, 12]. In our previous studies in Ethiopia, depression during pregnancy was associated with the increased use of emergency healthcare [13], unplanned (emergency) institutional delivery [14] and increased perinatal complications [15].

As part of its mental health Gap Action Programme (mhGAP), the World Health Organization (WHO) has identified evidence-based packages of care for key mental health conditions that can be delivered by non-specialist healthcare providers in low- and middle-income countries (LMICs) [16]. These care packages include recommendations for brief psychological interventions, including interpersonal therapy (IPT), cognitive behavioural therapy (CBT) and problem-solving therapy (PST) [17–20]. Although evidence suggests that women with perinatal depression can be treated effectively with psychological interventions delivered by non-specialists [17–20], research to adapt or develop such psychological interventions for rural, low-income African settings is limited.

In Ethiopia, mental healthcare remains highly centralised. Mental health specialists are located in urban hospitals, whereas over 80% of the population lives in rural areas. As the focus of the specialist mental healthcare is on severe mental illnesses, such as schizophrenia and bipolar affective disorder, there is a large treatment gap (96% of women had no access to treatment) for perinatal depression [21]. Expanding access to mental healthcare for women with common mental disorders, such as depression, is nationally underway through the integration of mental health into primary healthcare (PHC) and maternal care services [22]. However, efforts to improve perinatal mental health are hampered by a lack of contextually relevant and scalable therapeutic options.

The importance of tailoring existing interventions for the new context where they are to be delivered is increasingly acknowledged [23]. Several studies conducted in Ethiopia have investigated perinatal depression in terms of risk factors [24], its public mental health impact [1, 10, 13–15, 25–27], the large treatment gap [21, 28], and women’s coping strategies and help-seeking preferences [21], but to date, there has been limited emphasis on development or adaptation of therapeutic options. The aim of this study was to describe the process by which we selected and adapted a brief psychological intervention to meet the needs of women experiencing antenatal depression in rural Ethiopia.

## Methods

### Context

This study was conducted in two purposively selected primary healthcare (PHC) facilities in Sodo district of the Gurage Zone of the Southern Nations, Nationalities and People’s Region (SNNPR) in Ethiopia. Sodo district is located 103-km south of Addis Ababa and comprises 58 predominantly rural sub-districts (*kebeles*) [29]. The official language of the region is Amharic, and the main livelihood is agriculture. In Sodo, there is one primary hospital (serving a population of around 160,000) and eight health centres (serving around 25,000 households each). Each health centre is linked to 3–5 health posts, which are grassroot level healthcare facilities where health promotion and illness prevention activities take place. Each health post is staffed by female community-based health extension workers (HEWs) who are responsible for maintaining a list of pregnant women in their catchment area. HEWs work to support the engagement of pregnant women in antenatal care in collaboration with the women’s development army (WDA), a network of volunteer women for healthcare promotion. One WDA volunteer is assigned to every 25 women in the community. Routine antenatal care is delivered within health centres and the primary hospital, with referrals of high-risk pregnancies from health centres to the primary hospital. In line with the National Mental Health Strategy of Ethiopia [22], most of the midwives, nurses and health officers at these facilities had previously received training in the WHO mhGAP intervention guide [16] and were supported to deliver first-line mental healthcare.

### Selecting and adapting an intervention model

To select and adapt a brief psychological intervention from existing evidence-based interventions, we followed new Medical Research Council (MRC) and National Institute of Health Research (NIHR) guidance for the development and evaluation of complex interventions [30]. The MRC/NIHR guidance recommends a phased process focused on (1) development, (2) feasibility testing, (3) evaluation, and (4) implementation.

We opted to use the ADAPT-ITT approach [31] to accomplish the first two phases of the MRC/NIHR framework since ADAPT-ITT had important elements to engage stakeholders. ADAPT-ITT comprises eight steps. The first two steps focus on pre-adaptation activities, including the assessment of stakeholder needs and the selection and modelling of an evidence-based intervention. The remaining steps of the ADAPT-ITT approach focus on adapting the selected evidence-based intervention. We used the ecological validity model (EVM) [32] to guide our adaptation of the intervention’s content. The EVM prioritises adapting intervention content by modifying: language, persons, metaphors, concepts, goals, methods and context.

### Pre-adaptation work to select and model the intervention

We conducted formative qualitative research to explore the perspectives of women and healthcare workers (HCWs) on antenatal mental health and well-being, self-care activities and existing help-seeking and intervention preferences, as well as the potential acceptability of psychological interventions in the rural Ethiopian context. Participants in the formative qualitative study were potential intervention providers (*n*=8) health centre-based primary care workers and midwives, community-based HEWs (*n*=7) and potential service recipients (pregnant women, *n*=8). A detailed description of this study is published elsewhere [33].

The formative study found that women and HCW participants conceptualised women’s emotional problems in terms of local idioms, such as ‘thinking too much’ and linked them to social adversities [34]. HCWs expressed positive views towards providing a brief intervention for women with antenatal depression. HCWs reported that they did not formally assess women for antenatal depression, although most felt confident to ask about depressive symptoms. PHC staff stressed that detecting depression depended on gaining women’s trust, emphasising confidentiality and professional ethics and asking about social adversities before raising mental health matters. Both women and HCWs preferred the healthcare setting for intervention delivery, due to privacy concerns related to stigma. Potential barriers to intervention implementation included women’s domestic and occupational workloads, and their limited engagement with antenatal care, the overloaded healthcare system and diverse views about mental health [34].

**Fig. 1:**
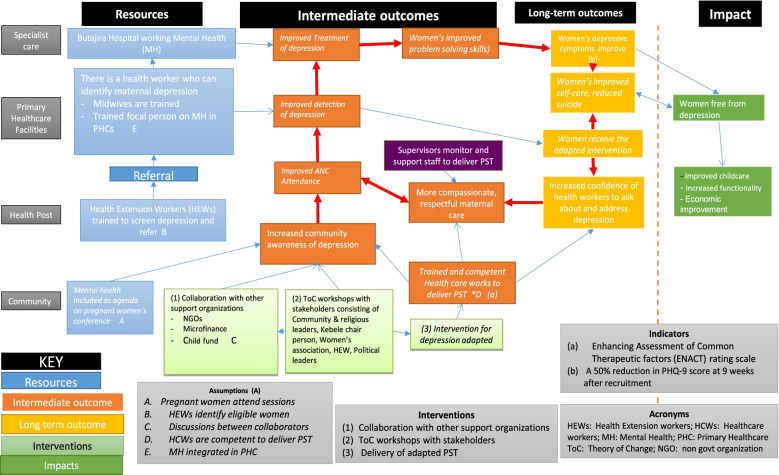
Theory of change map

To explore how brief psychological interventions could achieve the desired benefits for women and would fit into the antenatal care system, we employed a theory of change (ToC) approach [35]. Our goal in employing ToC was to develop consensus among stakeholders about the steps required (a ‘roadmap’) to improve the mental health of pregnant women in this rural Ethiopian setting. Through a series of ToC workshops, we identified intermediate steps required to achieve this goal, activities required to achieve each step, the rationale for such activities, underlying assumptions, and indicators of success. Three ToC workshops were attended by 24, 15 and 9 participants each. The first ToC workshop was attended by HCWs, mental health experts, researchers, community members, health planners, district health facility managers, representatives from the Youth and Children’s office, Social and Labour Affairs office, members of the WDA, non-governmental organisations and traditional healers. HCWs, mental health experts, researchers, district health facility managers and WDA members attended the second ToC workshop. The attendees of the third ToC were researchers and PHC staff. Minutes were taken at each workshop, to capture discussions before we produced a ToC map (Fig. 1).

The consensus of the three ToC workshops was that the ultimate impact of the intervention was for women to be free from depression, able to care for their child and function well. The long-term outcome was to improve women’s depressive symptoms and self-care and to reduce suicide through the adapted intervention. The psychological intervention was anticipated to have two effects: (1) to improve women’s functioning and (2) to increase HCWs’ skills to communicate about and detect perinatal depressive symptoms. Alongside the psychological intervention, it was expected that stakeholder collaboration during the implementation of the intervention would improve community awareness about perinatal mental health conditions, leading to increased antenatal care attendance, better communication between women and HCWs and increased reporting of mental health concerns to HCWs to improve treatment effects. Assumptions underlying the steps of the ToC were that HEWs would be competent to identify women and refer, pregnant women would attend psychological intervention sessions, stakeholder buy-in would be achieved, andHCWs would be competent to deliver PST and that there would be a functioning mental health platform, integrated into PHC.

We selected a South African model of brief problem-solving therapy (PST) [36] as the intervention to be adapted for the Ethiopian context for several reasons. These included the problem-orientated nature of stakeholders’ perceptions of Ethiopian women’s psychosocial distress in primary care [26], the association of perinatal depression with impaired problem-solving and coping [27], and the simplicity and acceptability of the therapeutic model for a low-literacy population [37–41].

### The nature of PST

PST assumes that low mood and problem-solving are interconnected, with low mood impairing problem-solving skills and coping. Reduced problem-solving and coping in turn lower the person’s mood further worsening symptoms of depression and anxiety.

PST focuses on improving a person’s ability to cope with problems and stressful life experiences [39]. PST has three phases: first, identifying the most important things in the person’s life and second, listing and categorising all problems into those (i) that are not important (Group A), (ii) that are important but cannot be solved (Group B) and (iii) that are important and can be solved (Group C). The third phase is making a plan to address each group of problems, to achieve what the person considers to be the most important things in their life. In meta-analyses, PST has been found to treat depression [40, 41]. PST has been found to be effective and acceptable in clinical populations with relatively low levels of education [37, 38, 42, 43]. We selected the South African version of PST due to its cultural and contextual relevance to our rural Ethiopian setting.

### The south African PST intervention model

In South Africa, a three sessions PST with one booster session combined with motivational interviewing (MI-PST) was adapted to reduce alcohol use disorders and depression in a range of in a range of clinical settings [36, 44, 45]. The first session lasted for 60 min, and the subsequent sessions lasted for 40 min [36]. The authors demonstrated the acceptability of MI-PST and the efficacy in reducing substance use and depression [46, 47]. In this version of PST, the MI component was included to help to improve motivation for care and thereby rates of treatment retention. MI-PST-trained professionals provided participants in this higher-literacy setting with a text booklet that summarised the session content and allowed participants to write their personal logs during sessions. The MI-PST was delivered face to face in the primary healthcare setting by peer counsellors having a bachelor-level education [36].

### Adaptation of MI-PST

We used ADAPT-ITT [31] to modify MI-PST for this rural Ethiopian context and used the ecological validity model (EVM) [32] to consider its contextual fit.

#### Step 1. Training Ethiopian mental health experts in MI-PST and initial adaptation

A trainer from the original MI-PST group provided a 5-day face-to-face training course to 17 technical experts from Ethiopia (psychiatrists, PhD students, master’s degree-educated mental health professionals, clinical and counselling psychologists). The training was conducted in Addis Ababa. Following the training, we conducted a full-day adaptation workshop with course attendees. We presented findings from the qualitative formative interviews and ToC workshops, discussing potential challenges and opportunities for the delivery of the proposed intervention, and recommendations for content modification. All feedback was documented. RK, and TB had a series of face-to-face and online meetings to accommodate stakeholder feedback on the PST intervention manual, which was also shared with senior members of the team. The output of this workshop was the first version of the intervention manual, which was then translated into Amharic (version 1.0).

#### Step 2. Theatre testing

Theatre testing is a structured, collaborative approach [31] for culturally adapting an intervention that forms part of the ADAPT-ITT approach. It exposes the stakeholder groups to the intervention model and its content by participating or viewing a mock demonstration. Afterwards, the research team and participants engage in a detailed critique of intervention content and delivery. Version 1.0 of the adapted intervention was presented to two groups of stakeholders using theatre testing [31] in the study site area. A clinical vignette was developed for the demonstration. A clinical psychologist (demonstrating the therapist’s role) and a mental health professional (demonstrating the woman’s role) role-played the intervention sessions to two groups of stakeholders: (1) professionals (psychologists, mental health experts, PHC clinicians and psychiatrists) and (2) women (six perinatal women with experience of depressive symptoms and two members of the WDA). At the end of each demonstrated session, two facilitators led separate group discussions with each of the two groups of stakeholders. These addressed aspects of the intervention that were most salient, parts that required revision and suggestions for improving the relevance and acceptability of the content. After the final session demonstration, the entire intervention model was discussed, and discussions were feedback recorded in minutes. TB and RK then collaborated to translate theatre-testing feedback to further modifications, leading to version 2.0 of the adapted PST.

#### Step 3. Local and international technical input

Version 2.0 of the manual was then reviewed by a diverse team of local and international experts. The team comprised three mental health professionals and researchers from South Africa (KS, BM, SH), two from the UK (RK, CH) and one from Ethiopia (TB). Suggestions and recommendations were integrated into version 3.0 of the intervention manual.

#### Step 4. PHC staff input

To obtain feedback on version 3.0 of the intervention from likely end-users, we trained 12 PHC staff (health officers, nurses, and midwives) from two health centres in the Sodo district. These cadres were identified by stakeholders as being best placed to deliver the adapted intervention during pre-adaptation formative qualitative interviews and ToC workshops. The training programme consisted of (1) 5 days of classroom-based training and (2) accelerated delivery of four intervention sessions to a first clinical case, using high-intensity supervision and feedback to build competence. The training focused on basic counselling skills, depression during pregnancy and practising brief PST using role plays and peer feedback. Accelerated delivery meant that each trainee delivered the intervention to one pregnant woman with depressive symptoms over a 2-week period. Barriers and challenges to the delivery of the intervention were noted, and PHC staff were approached to provide feedback and recommendations on the intervention content and delivery through qualitative methods as reported in the trial protocol [48]. Although the COVID-19 pandemic delayed the commencement of training, the course itself was designed to adhere strictly to the Ministry of Health guidelines. Likewise, delivery of PST sessions followed a standard operating procedure reflecting the need for physical distancing, hand hygiene and wearing of masks at all times. The final reporting of this intervention development was guided by the Template for Intervention Description and Replication (TIDieR) [49], a template for better reporting of intervention development and Standards for QUality Improvement Reporting Excellence checklist (SQUIRE) [50]. Please see Additional File for checklists.

## Results

A summary of the adaptations resulting from each step of the adaptation process is provided in Table 1. We have also described the details for each of the steps and the adapted EVM dimensions in each of the steps in the text below Table 1.

**Table 1:** Steps, adaptations and outputs

Adaptation steps	Adaptations	Outputs
1. Training of clinical stakeholders in MI-PST	Adaptation of terminology and inclusion of culturally relevant case examples while keeping a four sessions MI-PST along with the booster session. Pictorial flipchart added for the low literacy setting. Addition of strategies for facilitating women’s engagement such as training PHC workers in basic communication skills and empathetic care and relaxation exercises to increase PHC staff competence. The resultant version was then translated into Amharic.	
2. Theatre testing of version1.0 with key stakeholders	Renaming problem categories to be more culturally congruent. Including more exercises to demonstrate coping strategies for different problem categories. Some content from the second session was moved to the third session after feedback from theatre testing.	
3. Obtain local and international technical input on version 2.0	MI components were dropped due to cultural incongruence its specific use for alcohol.	
4. Obtain input from PHC staff on version 3.0	Half day refresher training was delivered, and as training PHC workers in helping women control their emotions.	

### Training of Ethiopian technical experts in MI-PST (step 1)

In the initial adaptation workshop, the key areas of adaptation were as follows:

#### The EVM domain of content

**Fig. 2:**
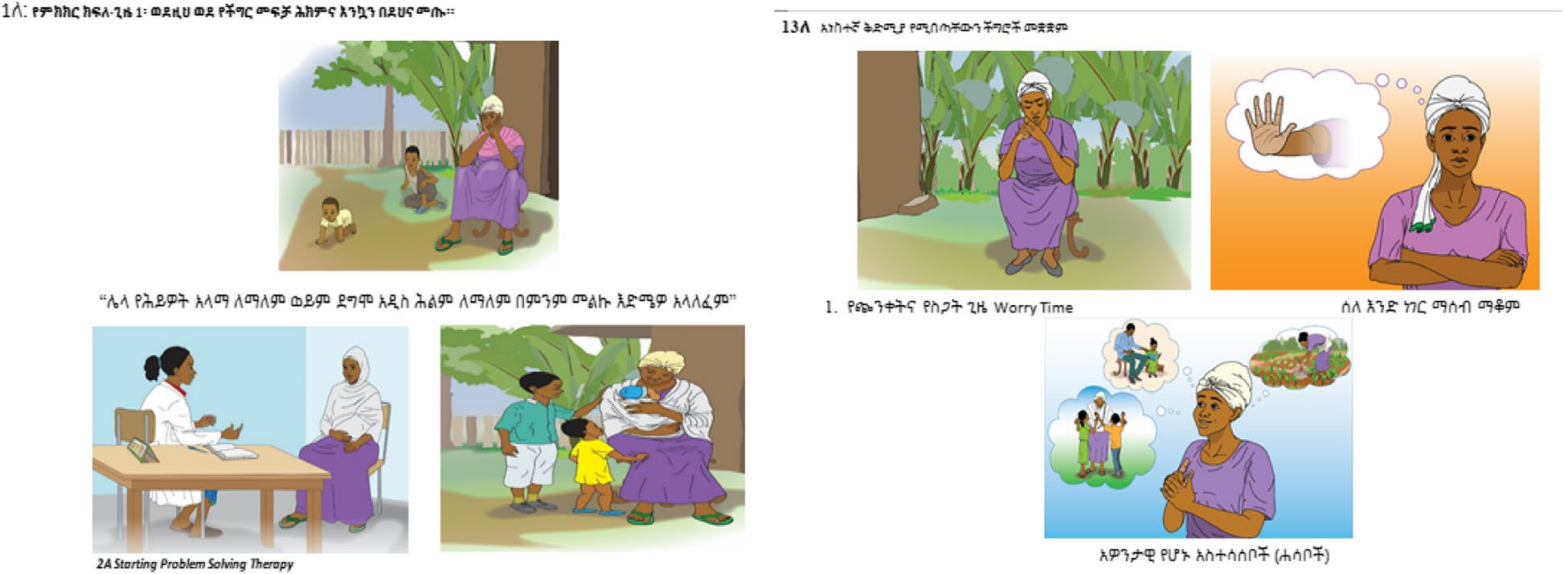
Model illustrations included in the flipchart

We modified some of the terms used by MI-PST for the Ethiopian context. For example, adaptation workshop attendees found that group A problems being termed ‘not important’ was problematic in the cultural context because all problems were considered important. Following extensive discussion, letters A, B and C were removed, and problem categories re-named as ‘lower priority problems’, ‘problems that cannot be changed’ and ‘problems that can be changed’. The verb ‘change’ was adopted instead of ‘solve’ because the stakeholders emphasised that women following PST might be unable to fully solve their problems. We replaced the personal log with a session record for the PHC worker to complete, while adding a pictorial flipchart (Fig. 2) to address concerns regarding comprehension in a low-literacy population.

#### The EVM domain of methods

We adapted session details, reduced the duration of the first session from 60 to 40 min, increased the number of sessions from three to four and selected the PHC setting for women’s privacy and more feasible delivery. We introduced strategies to facilitate women’s engagement such as training PHC workers in the importance of showing empathy during sessions and stress management strategies (relaxation exercises) to the intervention manual.

#### The EVM domain of concepts

We used culture-specific stakeholder perspectives and treatment preferences and existing coping mechanisms for depressive symptoms that we identified in the formative qualitative study to develop scripts of illustrative cases for use during intervention training. We developed pictorial flipcharts in collaboration with a local illustrator to assist women with limited education to follow PHC staff instructions during each session.

#### The EVM domain of context

MI-PST comprised three sessions and one booster session. Our Ethiopian adaptation entailed four sessions, fitting with the minimum number of antenatal care appointments recommended for pregnant women in this setting.

### Adaptations from theatre testing version 1.0 (step 2)

#### The EVM domain of goals

Pregnant women at the theatre testing expressed that role plays reflected the realities of their lives and praised the potential for PST to enable women to manage their problems and think about their lives. They expressed their pleasure at observing health workers showing concern for women’s well-being. ‘if the professional can listen to women, there is nothing that they won’t talk about, including sexual affairs’ a professional participant).

#### The EVM domain of concepts

Identifying the most important things in life was considered culturally relevant to women. However, all theatre-testing participants (women, PHC and mental health professionals) struggled to group problems into three categories. The training manual was modified to remind trainees to support women to take the lead in categorising their own problems. This was due to concerns that PHC workers may otherwise seek to problem-solve on women’s behalf. The need to emphasise this point in fidelity checks, and supervision was noted and included in the trial protocol [48].

#### The EVM domain of methods

Session two addressed all three types of problems and was felt by the stakeholders to have too much content. To address this, PST content was broken down into sessions using a session record form, held by the counsellor, with content broken down for the participant using the flipchart as a visual aid. We moved ‘problems that cannot be changed’ to the third session. Thus, our adapted version of PST provided an overview of the intervention in the first session. The second and third sessions then focused on ‘lower priority problems’ and ‘problems that cannot be changed’. In the final session, the counsellor reviewed progress, reinforced what was learned and worked on another ‘problem that can be changed’.

Theatre-testing participants expressed concern that women might not disclose their real problems and instead seek rapid solutions for less important problems. They also queried whether women might expect financial or other types of support alongside talking therapy. Others felt that women’s engagement would be determined by counsellors’ empathy, such as praise and affirmation, patience and careful introduction of the problem-solving approach. Most women and some professionals thought that women would disclose their problems, as long as confidentiality was assured (‘nothing is going to be hidden from a health worker and from a lawyer or from God/Allah’, woman discussant.

Theatre-testing participants also expressed their concerns that the screening tool for depressive symptoms (the Patient Health Questionnaire (PHQ-9) [51]) overlapped with women’s main problems or worries and took longer than expected to administer. As a result, concerns were expressed that women might not wish to stay for the full duration of the PST session. Other suggestions from theatre-testing participants included the need for more intensive training on counselling skills and specifying goals to promote clarity intervention contents.

Women participants appreciated PST sessions as they offered women time and space to think and share ideas. This supported the need to develop PHC staff counselling skills of communication and developing therapeutic rapport. We therefore increased the emphasis in PST training on basic counselling principles. Comments from theatre-testing participant discussions were included in the second draft of the intervention material (version 2.0).

### Local and international technical input (step 3)

#### The EVM domains of context and concepts

The MI elements in the original South African version of MI-PST were anticipated to support women’s engagement in PST. Indeed, we initially adopted a blended model of MI and PST because it appeared to fit the context well. However, a team of experts attending the adaptation workshop, professional participants in theatre testing and reviewers of the manual considered the MI component difficult to understand in this context. It was therefore removed from the manual.

### PHC staff input on version 3.0 (step 4)

#### The EVM domain of content

The supervisor assessment of PHC staff competencies after attending training identified gaps in basic communication skills and empathetic care. Half a day of refresher training was therefore delivered to counsellors to increase their competence in these areas.

#### The EVM domain of persons

The supervision of accelerated cases was an opportunity for PHC staff delivering PST sessions to discuss practical challenges with their trainer—supervisors. However, developing counsellors’ competence to support women to list their life problems was not straightforward. Some PHC staff reported that some women reported having no problems in session one, preventing subsequent intervention steps from being followed. We noted the importance of including the trial standard of operating procedures (SOP) about strictly supervising whether counsellors were adequately discussing the purpose and privacy of audiorecordings that were supposed to facilitate supervision. Due to limitations in counsellors’ communication skills and adherence to the manual, supplementary training was delivered on controlling women’s emotions with refresher training content. The final adapted version of the intervention has four face-to-face sessions to be delivered by PHC staff to antenatal women. The first session lasts for 40 min and the remaining sessions last for 30 min. The final adapted version of intervention will be electronically available. It involves provider training manual, pictorial flipchart, session record and leaflet.

## Discussion

We followed a series of iterative procedures, with input from multiple stakeholders, selecting and adapting PST to optimise a brief psychological intervention for women experiencing perinatal depression in rural Ethiopia. Adapting an evidence-based psychological intervention for this context was crucial [52] to address the gap between the need for and provision of perinatal mental healthcare in this low-income setting [21, 28].

Our pre-adaptation work, including a formative qualitative study [34] and theory of change workshops, enabled us to identify PST as the most appropriate evidence-based intervention for our rural Ethiopian setting, given the relationship between depression and social adversities. Women perceived depressive symptoms (“thinking too much”) as a reaction to social problems. PST aims to help people with depressive and anxiety symptoms to restore and improve their problem-solving and coping skills in order to break the bidirectional link between problems and depressive symptoms. Our intervention selection also cohered with evidence from LMICs supporting the effectiveness of psychological treatments for perinatal depression that focus on social problems [17, 18] and are delivered within routine maternal and child healthcare [20].

Interpersonal therapy (IPT) and cognitive behavioural therapy (CBT) were also suggested in the WHO mhGAP intervention guide [16], for treatment of depressive disorders. Qualitative interviews and focus group discussions after an uncontrolled, non-randomised pilot study [53] suggested that IPT was feasible to deliver and acceptable to participants with HIV in Ethiopia. However, the authors acknowledged that social desirability bias could have affected participant feedback, although data collectors were independent of the intervention. On the other hand, CBT-based approaches, such as the thinking healthy programme, assume a certain level of literacy and their intensity (length and number of sessions) may not be feasible for women in rural, low-income settings. We adapted a four-session brief PST supported by pictorial flipchart for non-literate low-income rural women.

Finally, we adapted the intervention content, language, case examples, illustrations, exercises and homework tasks to align with women’s and counsellors’ perspectives and preferences [33]. For example, we replaced the woman-held, text-dominated intervention booklet tailored for South Africa with a pictorial flipchart, to increase confidentiality and acceptability in this low-literacy setting. We also adapted the methods, context and goals of the intervention, including the approach to counsellor training and supervision and duration of treatment. Women and PHC staff’s recommendation that PST should be delivered by antenatal care providers accorded with findings from previous studies [19], where PST delivered by healthcare workers within routine maternal and child healthcare was found to be effective. Our recruitment of antenatal staff as PST counsellors was also consistent with the adaptation of PST in India [54]. However, our counsellor recruitment differed from that of the Friendship Bench in Zimbabwe [37], a model of PST delivered by volunteer grandmothers. This model was unsuited to our context due to concerns about the feasibility and sustainability of relying on unpaid workers, challenges in their interface with the formal healthcare system and potential stigma, all of which were concerns raised in our preadaptation qualitative study [33].

Each adaptation aimed to reduce barriers that could undermine acceptability, related to context, language, counsellor-related barriers (such as motivating and facilitating trust) or participant factors (such as expectations of the intervention). This approach was in line with necessary adaptations reported in a systematic review and qualitative meta-synthesis of process evaluations of task-sharing interventions for perinatal depression in LMICs [18]. During pre-adaptation work, we also identified women and PHC staff perspectives on antenatal depression in rural Ethiopia. Understanding such perspectives [55–57] and idioms of mental illness [58] are key aspects of adapting evidence-based interventions.

The motivational interviewing component in the original South African version of the MI-PST was originally included to improve participants’ motivation to access care, and thereby, rates of treatment retention in the context of alcohol use [36]. Although potentially applicable to increasing engagement in the context of depressive symptoms, the techniques that MI employs to improve motivation were less relevant and more difficult to implement for women with depressive symptoms in our setting. Our adaptation to include a pictorial flipchart due to the low literacy setting and addition of foundational communication skills are distinctive modifications to address commonly recognised implementation barriers [18]. Furthermore, we employed accelerated cases and supportive supervision to enhance PHC staff competence: a fundamental barrier to the efficacy of psychological interventions noted in low-income settings [18]. However, competency assessments identified a need for additional training in responding to women in acute distress, consistent with previously-identified challenges of task-shared mental health interventions [18].

Our final, the adapted version of PST was a four-session, individual, face-to-face intervention to be delivered by PHC. All adaptations to the intervention materials involved prospective service users and counsellors, without altering the core elements of the original intervention [59]. However, the findings should be understood in light of several limitations. The intervention was adapted in the rural Ethiopian context, and its feasibility and acceptability may differ in other low- and middle-income country contexts. Given the paucity of interventions for perinatal mental health problems in low-income countries, however, this adapted intervention can provide a foundation for other investigators to further adapt to specific contexts and communities. In the context of too centralised and inaccessible perinatal mental healthcare services, integrating perinatal psychological interventions within antenatal care can help to improve the quality of psychosocial care for all women. This integrative approach, once tested for its feasibility and acceptability, equips health workers (midwives) with communication and counselling skills that facilitate woman-centred care and can help to ensure that mental health is seen as a key component of maternal health.

## Conclusion

Using a rigorous, iterative and participatory process, we selected and adapted brief problem-solving therapy to meet the needs of pregnant women with depressive symptoms in a rural, low-literacy Ethiopian context. The next step will be to assess the feasibility and acceptability of intervention delivery within routine antenatal care settings through a pilot randomised trial.

## Supplementary Information

**Additional file 1.**

## Data Availability

Not applicable.

## Abbreviations

MRC: Medical research council
HICs: High-income countries
SDG: Sustainable development goals
LMICs: Lower middle-income countries
PHC: Primary healthcare
PHQ-9: Patient Health Questionnaire-9
PST: Problem-solving therapy
RCT: Randomised controlled trial
SRQ-20: Symptom Reporting Questionnaire-20
