# Supplementary material for: Adapting an intervention of brief problem-solving therapy to improve the health of women with antenatal depressive symptoms in primary healthcare in rural Ethiopia
Source: Pilot Feasibility Stud. 2022 Sep 9;8:202. doi: 10.1186/s40814-022-01166-1 (PMC9461178; doi:10.1186/s40814-022-01166-1)
Supplement: Supplementary file 1 — Additional file 1. [file 40814_2022_1166_MOESM1_ESM.zip › TiDER_Intervention devt form - cleanR2.docx]

## Intervention Development form (Modified version of the MRC Complex Intervention Guidance (Hoddinott, adapted further for ASSET)

When should this be completed – once at the beginning, and once at the end of the intervention development process

| QUESTION | RESPONSE |
| --- | --- |
| Name of intervention | Problem-Solving Therapy (PST) and PST adapted for women experiencing intimate partner violence (IPV; PST-IPV) |
| Version | Initial |
| 1a. What are you trying to do? | - Equip PHC workers with the skills and confidence to discuss women’s mental health and social difficulties such as IPV. - Equip PHC workers with skills to deliver brief PST for depression. - (PST-IPV only) Train PHC workers to respond to disclosures of IPV within antenatal care (ANC). |
| 1b. What outcome are you aiming for? | - More holistic antenatal care, which addresses women’s psychosocial wellbeing in addition to their obstetric health. - (Ultimately – cannot be demonstrated by feasibility trials) Improved antenatal common mental disorder (depression, anxiety, post-traumatic stress disorder (PTSD)) symptoms and functioning. - Better detection and treatment of psychosocial problems (IPV and common mental disorder symptoms) in perinatal women. - (PST-IPV only) Better responses by PHC workers to disclosures of IPV within ANC. |
| 1c. How will you bring about the change? (please highlight where the intervention seeks to achieve or promote behavioural change) | - PHC workers are confident to proactively explore the woman’s emotional health and psychosocial status [behaviour 1] - PHC workers have skills to respond to women’s distress, follow the SOP guidance for the ‘distressed/traumatised patient’ and implement an appropriate management plan [behaviour 2] - PHC workers are trained to deliver evidence-based brief PST for antenatal depression and apply these skills in appropriate research contexts (where women have been randomised to this arm of the feasibility trial) [behaviour 3] - (PST-IPV only) PHC workers are trained to respond to disclosures of IPV within ANC [behaviour 4]. - PHC workers are supported to deliver PST or PST-IPV, with appropriate safeguards and regular supervision, including discussion of onward referrals for at-risk women [behaviour 5]. |
| 2. What is the theoretical basis for your intervention? | - the intervention is based on the Carl Rogers interpersonal relations theory and Peplau’s theory of Nurse-Patient Relationship. |
| 3. How have you used this theory in the development of the intervention? | PHC workers will be trained to about basic counselling skills focusing on establishment of good rapport or relation with patients and CRC techniques. They will also be trained about basic communication skills |
| 4. Have you described the intervention fully according to TIDieR guidelines, so that it can be implemented properly for the purposes of your evaluation and replicated by others? | PST and PST-IPV descriptions are provided in individual study protocols soon to be submitted for peer-reviewed publication. |
| 5a. What existing evidence (ideally collated in a systematic review) supports the development of your intervention so that it is likely to be feasible, effective and cost effective? | - ASSET scoping review of non-technical skills for health workers - Diagnostic phase evidence/previous research evidence from Ethiopia showing low level of person-centred care (PACIC, AFFIRM-ENACT) and low detection of psychosocial problems in PHC and maternal care (PRIME). - There is growing evidence for the effectiveness of brief, task-shared psychological interventions for common mental disorders (CMDs) such as depression and post-traumatic stress disorder (PTSD) in LMICs; meta-analysis of 27 RCTs found a pooled effect size of 0·49 (Singla et al., 2017). - A meta-analysis of PST for depression found a standardised mean effect size of 0.34, but high heterogeneity among included studies was not explained by sub-group analyses, indicating the need for research into the settings and patient groups for whom it is effective (Cuijpers et al., 2007). - A meta-analysis of PST for any mental or physical health problem found that it was significantly more effective than no treatment, treatment as usual, and ‘attention placebo’ arms (controlling for non-specific effects of contact), moderated by the use of problem-orientation training and homework assignments (Malouff et al., 2007). - Several randomised controlled trials from LMICs suggest that PST can be effective for treating depression, anxiety and psychological distress (Sorsdahl et al., 2015; Chibanda et al., 2016). |
| 5b. To what extent can this evidence be generalised to your context? | - The available evidence applies to low-income (e.g. Zimbabwe) and middle-income settings (e.g. South Africa) which share some contextual challenges relevant to rural Ethiopia. - However, few studies have explored PST in low-income settings with the resource restrictions of rural Ethiopia. This justifies the adaptation of existing interventions to meet the specific cultural and contextual needs of this setting. |
| 6. What steps have you taken to a) Enhance the implementability of the intervention in your context and b) Promote wider scalability in the region? | a) The implementability of PST and PST-IPV is enhanced by situating them within ANC, where PHC workers have been trained using the Ethiopian Primary Healthcare Guideline (PHCG) platform of facility-based, team-based training and close collaboration. PHC workers provided feedback on the PST intervention model during theatre testing which took place during the adaptation phase.  b) Both PST and PST-IPV are first being evaluated using feasibility trial methods, to ensure that their implementability and scalability in the region are explored at the earliest stage, permitting adaptation and optimisation in response to detailed evaluation. |
| 7. Are you aware of any potential biases in the intervention development process, and what steps have you taken to avoid them, or mitigate their effects? | We have obtainedd broad stakeholder input:   1. Two Theory of Change workshops brought together district health office planners, community elders, multidisciplinary representatives, ANC primary healthcare workers (including health extension workers, midwives, nurses), mental health professionals, and a psychologist involved in the development of PST’s original materials. 2. Topic experts including those with clinical and research experience of implementing PST and other models of brief talking therapy in South African ANC contributed to intervention development. 3. Both PST and PST-IPV have been adapted in response to a set of in-depth qualitative interviews with women and PHC workers about the acceptability, feasibility and need for psychosocial interventions in ANC which address social difficulties like IPV. |
| 8. Please provide 3 level EPOC classification codes to taxonomise the main features of your intervention | 3.3.1 Role expansion or task shifting  3.3.2 Self-management  3.4.7 Disease management  3.4.8 Integration  3.4.14 Shared decision-making |
| Lavis | 3.2.7 Skill mix – role expansion or extension  3.2.12 Staff – Training  3.2.13 Staff – Support  3.2.16 Staff – Shared decision-making  3.2.17 Self-management  3.3.4 Integration of services  [have not coded the Lavis implementation strategies] |

## 2. Modified set of Theoretical Determinants Framework questions

If the intervention includes an element of desired behaviour change then complete the set of TDF questions below.

When should this be completed? – For each targeted behaviour, once at the beginning, and once at the end of the intervention development process

Table – TDF suggested specification of targeted behaviour (14)

| Question | Description |
| --- | --- |
| Name of intervention | Maternal Care Clinical Communication Skills |
| 1. What is the behaviour that you want to change? | 1. PHC workers are confident to proactively explore the woman’s emotional health and psychosocial status [behaviour 1]  2. PHC workers have skills to respond to women’s distress, follow the PHC guidance for the ‘distressed/traumatised patient’ and implement an appropriate management plan [behaviour 2]  3. PHC workers are trained to deliver evidence-based brief PST for antenatal depression and apply these skills in appropriate research contexts (where women have been randomised to this arm of the feasibility trial) [behaviour 3]  4. (PST-IPV only) PHC workers are trained to respond to disclosures of IPV within ANC [behaviour 4].  5. PHC workers are supported to deliver PST or PST-IPV, with appropriate safeguards and regular supervision, including discussion of onward referrals for at-risk women [behaviour 5]. |
| 2. Why do you want to change it? Why is this a priority? | These behaviours are prerequisites to detect and respond to antenatal common mental disorder symptoms and exposure to intimate partner violence (IPV).  Both antenatal common mental disorders and IPV have been associated with poorer maternal and infant outcomes in this rural Ethiopian setting. |
| 3. Who is performing the behaviour? | The PHC worker involved in antenatal care (midwife, nurse, health officer). |
| 4. What do they need to do differently? (In what direction do you want the behaviour to change?) | 1. Explore women’s mental health and psychosocial difficulties  2. Respond to women’s distress and implement an appropriate management plan, where needed.  3. Deliver PST, to improve women’s skills to solve their problems.  4. Respond to disclosures of IPV within ANC (PST-IPV only).  5. Access training, support and supervision, including referring at-risk women onwards. |
| 5. When do they need to do it? | During the feasibility trial, only for women randomised to the relevant treatment arm (PST or PST-IPV).  Generally, (1), (2) and (4) in all maternal care consultations. |
| 6. Where do they need to do it? | ANC clinical settings. |
| 7. How often do they need to do it? | Part of routine care. |
| 8. With whom do they need to do it? | Pregnant and post-partum women. |
| 9. Which TDF domains and components are most relevant (give codes) | 1. Knowledge  2. Skills (competence)  3. Social/professional role and identity  4. Beliefs about capabilities (perceived confidence)  7. Reinforcement  11. Environmental context and resources (time, space and adequacy of interventions for poverty/emotional problems/IPV)  12. Social influences  13. Emotion  ** |

## 3. The revised TIDieR checklist (as recommended in ‘Getting messier with TIDieR’ (11)).

When should this be completed? – At the beginning of intervention development, at the end (which for TIDieR should be the end of the evaluation process), and on every occasion in between when there is a noteworthy modification to document.

The recommended practice requires some further modifications to the forms, since some items are version specific. I am suggesting three versions

a. An initial form, completed at the very beginning of the intervention development process, and before piloting

b. Revision forms to be updated during the intervention development process, and before piloting

c. Revision forms to be updated during piloting/ evaluation phases

a. An initial TIDieR form, completed at the very beginning of the intervention development process, and before piloting

| TIDieR item | Brief Description | PST and PST-IPV |
| --- | --- | --- |
| TIDieR version ID | Version number and date | PST and PST-IPV_ID01Version 1.0 30/12/2019 |
| 1. Brief Name | Name or phrase describing the intervention. | Problem-Solving Therapy (PST) and PST adapted for women experiencing intimate partner violence (IPV; PST-IPV) |
| 2. Why | Rationale, theory, or goal of elements essential to intervention. | See intervention development form. |
| 3. What (materials) | Physical or informational materials used, and where they can be accessed. | - Printed training manuals, in-session ‘flip chart’ documents, simple information leaflet. - Documents recording session discussions, retained by PHC workers. |
| 4. What (procedure) | Procedures, activities, and/or processes used in the intervention, including any enabling or support activities. | PHC workers will receive one day training in PST or PST-IPV.  PHC workers will attend monthly supervision with a psychology masters-educated supervisor, in which challenging cases and session recordings are discussed.  Research assistant and Trial Coordinator will monitor progress of the trial, including confirming that trial paperwork is completed and that PST and PST-IPV sessions take place. |
| 5. Who provided | Background, expertise of provider, and training given. | PHC workers will be selected by the researchers in consultation with the facility and randomly allocated to receive PST or PST-IPV training. They will usually be a more senior or experienced clinician. |
| 6. How | Modes of delivery, delivered to group or individual. | PST and PST-IPV will be delivered to women on an individual basis. Staff training will take place in groups, using role play and case studies to demonstrate specific communications skills, as well as group brainstorming activities. |
| 7. Where | Type of location. | Sessions of PST and PST-IPV will be delivered in health centres (primary health care) |
| 8. When and How Much | Number of times, number of sessions, intensity and over what time period delivered. | PHC workers will receive two days’ training in either PST or PST-IPV, which will include refreshing of prior training on communication skills in maternal care, which they received as part of ASSET’s maternal care and communication skills work package. |
| 9. Tailoring | What, why, when, and how of planned personalisation/ adaptation. | The PST intervention model was developed originally for substance use in South Africa.  It has been necessary to adapt the content to (1) be relevant for national initiatives and health system reform in Ethiopia, (2) to recognize the different interpersonal communication styles used in Ethiopia around emotions and cultural perceptions (expression of emotion as harmful), and (3) to recognize that there are limited resources to address social adversities.  PST-IPV has been further adapted to address the complex needs and experiences of women affected by intimate partner violence. |
| 11. How well (planned) | If intervention adherence or fidelity is to be assessed, describe how and by whom, and if any strategies will be used to maintain or improve fidelity. | A checklist will be used to monitor coverage of key content during PST and PST-IPV sessions, by reviewing session paperwork completion and a random sample of audio-recorded sessions, both by independent assessors and during PHC worker supervision.  Adherence will be quantified by retention rates within the trials and duration of PST and PST-IPV sessions. |
| 12. How Well (role of context) | Outline the contextual factors that you believe may have an impact on delivery. | - Stigma in PHC workers towards common mental disorders. - Perceptions by PHC workers of inadequate or unrealistic referral pathways for emotional problems/IPV Cultural barriers to exploring emotions and IPV - Conflicting priorities in clinical settings preventing PST and PST-IPV from being prioritised - Understaffing or attrition of trained staff - Unforeseen geopolitical eventualities compromising continuity of PST and PST-IPV during the course of the second and third trimester |
| 13. Voice | Whose voice does this version of TIDieR convey?  Who was involved in the preparation of TIDieR, how they were involved in the intervention/their perspective (e.g. researcher, service deliverer, patient, etc.)? | PHC providers and women with experience of depression have been involved in the inception of PST (through in-depth qualitative interviews and two Theory of Change workshops) and in adaptation of PST through a ‘theatre testing’ workshop. Topic experts have additionally been consulted throughout the adaptation process. |
| 14. Stage | What stage of implementation does this TIDieR checklist cover? | Intervention adaptation |

# INTERVENTION 2

## Intervention Development form

(Modified version of the MRC Complex Intervention Guidance (Hoddinott, adapted further for ASSET))

When should this be completed – once at the beginning, and once at the end of the intervention development process

| QUESTION | RESPONSE |
| --- | --- |
| Name of intervention | Problem Solving Therapy |
| Version | Initial |
| 1a. What are you trying to do? | - Equip women with problem solving skills |
| 1b. What outcome are you aiming for? | - Improved problem solving skills and so positive affect among women (reduced depressive symptoms) |
| 1c. How will you bring about the change? (please highlight where the intervention seeks to achieve or promote behavioural change) | - Women will receive a 4 sessions PST; each session for about 40 minutes. - Women will be able to identify what they value most in their life - Women will be able to list and categorize their daily problems that obstacle attainment of their important things in their lives - Women will able to design strategies to solve problems and implement it. |
| 2. What is the theoretical basis for your intervention? | - it is based on CBT where women’s cognition about their daily lives affects their emotions and behaviours which are lined to depressive symptoms. It is based on well-established evidence that social adversities predict depressive symptoms and vice versa. |
| 3. How have you used this theory in the development of the intervention? | The intervention aims to teach women about problem solving skills so that they can manage their daily problems that are linked to their emotional problems |
| 4. Have you described the intervention fully according to TIDieR guidelines, so that it can be implemented properly for the purposes of your evaluation and replicated by others? | In process |
| 5a. What existing evidence (ideally collated in a systematic review) supports the development of your intervention so that it is likely to be feasible, effective and cost effective? | - There is systematic reviews demonstrating its efficacy |
| 5b. To what extents can this evidence be generalised to your context? | Substantial amount of the evidence highlighting the need has come from our context. |
| 6. What steps have you taken to a) enhance the implementability of the intervention in the context of your PSTAND and b) promote wider scalability in the region? | a) ToC done with stake holders  qualitative interviews conducted with women and health care workers to understand their perspectives on adapting psychological interventions. Theatre test conducted and comments received for changes on the manual and flipchart. The intervention suggested to be acceptable and feasible |
| 7. Are you aware of any potential biases in the intervention development process, and what steps have you taken to avoid them, or mitigate their effects? | 1. intervention initially emphasized on MI |
| 8. Please provide 3 level EPOC classification codes to taxonomise the main features of your intervention | 3.3.1 Role expansion or task shifting  3.3.2 Self-management  3.3.7 Disease management  3.3.7 Integration  3.4.14 Shared decision-making |
| LAVIS | 3.2.7 Skill mix – role expansion or extension  3.2.12 Staff – Training  3.2.13 Staff – Support  3.2.16 Staff – Shared decision-making  3.2.17 Self-management  3.3.4 Integration of services  [have not coded the Lavis implementation strategies] |

## 2. Modified set of Theoretical Determinants Framework questions

If the intervention includes an element of desired behaviour change then complete the set of TDF questions below.

When should this be completed? – For each targeted behaviour, once at the beginning, and once at the end of the intervention development process

Table – TDF suggested specification of targeted behaviour (14)

| Question | Description |
| --- | --- |
| Name of intervention | PST |
| 1. What is the behaviour that you want to change? | improved skill to solve problems so as to link with positive affect |
| 2. Why do you want to change it? Why is this a priority? | Because, daily problems are triggers and worsen emotional problems which again reduces their problem solving skills. This maintains both having daily problems and emotional problems on women. |
| 3. Who is performing the behaviour? | The PHC worker involved in antenatal care (midwife, nurse, health officer) |
| 4. What do they need to do differently? (In what direction do you want the behaviour to change?) | develop women’s skill to solve their problems |
| 5. When do they need to do it? | All maternal care consultations |
| 6. Where do they need to do it? | ANC clinic, PNC clinic, delivery room |
| 7. How often do they need to do it? | Part of routine care |
| 8. With whom do they need to do it? | Pregnant women |
| 9. Which TDF domains and components are most relevant (give codes) | 2. Skills (competence)  4. Beliefs about capabilities (perceived confidence)  7. Reinforcement  11. Environmental context and resources (time, space and adequacy of interventions for poverty/emotional problems/IPV)  12. Social influences  13. Emotion  Perhaps also 3. Social/professional role and identity?  ** |

## 3. The revised TIDieR checklist (as recommended in ‘Getting messier with TIDieR’ (11)).

When should this be completed? – At the beginning of intervention development, at the end (which for TIDieR should be the end of the evaluation process), and on every occasion in between when there is a noteworthy modification to document.

The recommended practice requires some further modifications to the forms, since some items are version specific. I am suggesting three versions

a. An initial form, completed at the very beginning of the intervention development process, and before piloting

b. Revision forms to be updated during the intervention development process, and before piloting

c. Revision forms to be updated during piloting/ evaluation phases

a. An initial TIDieR form, completed at the very beginning of the intervention development process, and before piloting

| TIDieR item | Brief Description |  |
| --- | --- | --- |
| TIDieR version ID | Version number and date |  |
| 1. Brief Name | Name or phrase describing the intervention. | PST |
| 2. Why | Rationale, theory, or goal of elements essential to intervention. | See intervention development form. |
| 3. What (materials) | Physical or informational materials used, and where they can be accessed. | - printed training manuals - pictorial representation of PST steps and stages - flip chart - video clip |
| 4. What (procedure) | Procedures, activities, and/or processes used in the intervention, including any enabling or support activities. | women will receive a four sessions PST or PST IPVs, |
| 5. Who provided | Background, expertise of provider, and training given. | The facility-based facilitator will be a primary health care clinician who has been selected by the researchers in consultation with the facility. They will usually be a more senior clinician who has some experience or role in management. The facility-based facilitator will be trained for 4-6 days by expert mental health professional |
| 6. How | Modes of delivery, delivered to group or individual. | individual-based. |
| 7. Where | Type of location. | Health centre (primary health care) |
| 8. When and How Much | Number of times, number of sessions, intensity and over what time period delivered. | 4 sessions each for 40-45 minites |
| 9. Tailoring | What, why, when, and how of planned personalisation/ adaptation. | The intervention was developed originally for substance use in South Africa.  It has been necessary to adapt the content to (1) be relevant for national initiatives and health system reform in Ethiopia, (2) to recognize the different interpersonal communication styles used in Ethiopia around emotions and cultural perceptions (expression of emotion as harmful), and (3) to recognize that there are limited resources to address social adversities. |
| 11. How well (planned) | If intervention adherence or fidelity is to be assessed, describe how and by whom, and if any strategies will be used to maintain or improve fidelity. | We will develop a checklist to monitor coverage of key content during the facility-based sessions. |
| 12. How Well (role of context) | Outline the contextual factors that you believe may have an impact on delivery. | - Stigma in PHC workers - Lack of adequate interventions for emotional problems/IPV or realistic referral pathways - Cultural barriers to exploring emotions |
| 13. Voice | Whose voice does this version of TIDieR convey?  Who was involved in the preparation of TIDieR, how they were involved in the intervention/their perspective (e.g. researcher, service deliverer, patient, etc.)? | - adapted from ASSET   PHC providers will be involved in the adaptation phase. |
| 14. Stage | What stage of implementation does this TIDieR checklist cover? | Intervention adaptation |
